# New FDA approved antibacterial drugs: 2015-2017

**DOI:** 10.15190/d.2018.1

**Published:** 2018-04-04

**Authors:** Stefan Andrei, Liana Valeanu, Radu Chirvasuta, Mihai-Gabriel Stefan

**Affiliations:** ^1^Intensive Care Unit, Centre Hospitalier Lyon Sud, Pierre Benite, France; ^2^Department of Cardiac Anesthesia and Intensive care, Emergency Institute for Cardiovascular Diseases “Prof. C.C. Iliescu” Bucharest, Romania; ^3^Carol Davila University of Medicine and Pharmacy, Bucharest, Romania; ^4^Anaesthetics Department, Lister Hospital, Stevenage, UK

**Keywords:** FDA approved drugs, ceftazidime, avibactam, obiltoxaximab, bezlotoxumab, delafloxacin, vaborbactam, vabomere, ozenoxacin, malacidin, teixobactin, 2015, 2016, 2017

## Abstract

Increasing bacterial resistance to antibiotics is a worldwide ongoing issue. Urgent need for new antibacterial agents has resulted in significant research efforts, with new molecules proposed for use in clinical practice. However, as highlighted by many groups this process does not have an optimal rhythm and efficacy, to fully combat highly adaptive germs, particularly in the intensive care units. This review focuses on the last three years of novel FDA approved antibacterial agents (2015-2017): ceftazidime/avibactam, obiltoxaximab, bezlotoxu-mab, delafloxacin, meropenem/vaborbactam, ozenoxacin. Ceftazidime/avibactam and meropenem/ vaborbactam are new players in the field of resistant bacteria treatment. Ceftazidime/avibactam is validated in selected patients with complicated urinary or intra-abdominal infections, hospital and ventilator-associated pneumonia. Meropenem/ vaborbactam gained approval for the cases of complicated urinary tract infections. Other potential indications are under investigation, widened and validated by future studies. Obiltoxaximab is a monoclonal antibody that can be used in the prevention and treatment of inhalational anthrax. Bezlotoxumab monoclonal antibody is an useful and specific tool for the management of recurrent Clostridium difficile infection. Delafloxacin is approved for patients with acute skin or skin structure infections. Despite recent progress, it is imperative to continue the development of new antibiotic drugs and new strategies to counteract resistance to antibiotics.

## 1. Introduction

Antibiotics discovery and clinical use is undoubtedly one of the pillars of modern medicine. Modern medicine saw a continuous competition between new antibacterial drug research and the ability of bacteria to develop resistance^1^. New classes of antibiotics were created, old drugs regained interest and paradigm changes were proposed^2-4^. However, this process seems to have diminished its pace and, after less than a century since the first clinical use of an antibiotic, bacterial resistance to antibiotics is a major concern of current medical practice and research^5^. The great influenza pandemics offers an unfortunate insight of a post-antibiotic era.

In the intensive care unit, dealing with resistant bacteria is a daily struggle. A careful antibiotic stewardship combined with public health prevention measures are vigorously promoted, in order to lower the incidence of resistant bacteria^2,6-9^. Despite sustained efforts, some bacteria highly susceptible to develop resistance, such as *Pseudomonas aeruginosa* or *Acinetobacter baumannii,* end up being treated by not so new drugs like colimycin^3,10,11^. From this perspective, the intensive care physician is looking regularly to the research field, expecting an ideal antibiotic: specific, effective, well tolerated and with no long term induced resistance.

In this present review, we briefly address the new antibacterial agents approved during the recent years by FDA, as a hope to reinforce the current therapeutic armamentarium. New antibacterial agents were identified using FDA (www.accessdata.fda.gov, www.fda.gov) and Center Watch sites (https://www.centerwatch.com/drug-information/fda-approved-drugs/).

**Figure 1:**
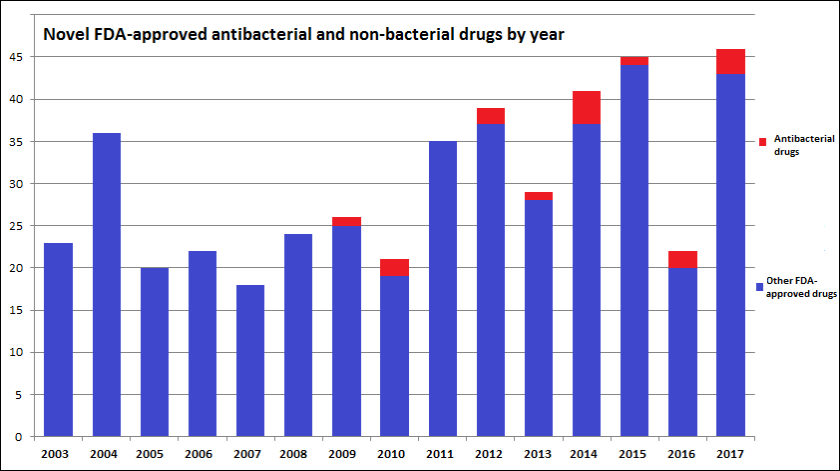
Novel FDA-approved antibacterial and non-bacterial drugs by year (last 15 years)

The number of FDA-approved antibacterial drugs and the total novel molecules for each year during the past 15 years is summarized in **Figure 1**. A tendency of increasing the number of approved molecules by year can be noticed. Furthermore, visibly more antibacterial molecules have been approved in recent years compared with previous years.

To the date of this review, no antibacterial drug has been approved for 2018, from a total of 6 newly introduced molecules. In 2017, the 3 approved antibacterial drugs represented 6.5% of the total of 46 new drugs. 2016 brought 2 new drugs, respectively 9% of the 22 approved molecules. In 2015, only one antibacterial agent was approved by FDA (2.2% from a total of 45).

We identified 6 novel FDA approved antibacterial drugs: the 3^rd^ generation cephalosporin and β-lactamase inhibitor combination ceftazidime/avibactam in 2015; two monoclonal antibodies, obiltoxaximab and bezlotoxumab in 2016; a new fluoroquinolone, delafloxacin, a combination of meropenem with the β-lactamase inhibitor vaborbactam, vabomere, and a non-fluorinated quinolone, ozenoxacin, all in 2017 (**Table 1**). These drugs are briefly discussed further by FDA-approval year.

## 2. FDA approved antibacterial drugs (2015-2017)

### ** *2.1 Ceftazidime/avibactam*

Ceftazidime/avibactam is a combination of ceftazidime, a third generation cephalosporin, with avibactam, a β-lactamase inhibitor^12^. Ceftazidime inhibits peptidoglycan synthesis by inhibiting penicillin-binding proteins, resulting in cell wall instability and cell death^13^. Avibactam is a synthetic non-β-lactam β-lactamase inhibitor that inhibits the activities of Ambler class A and C β-lactamases and of some Ambler class D enzymes, but not the B1 metallo-β-lactamases, such as the New Delhi metallo-β-lactamase (NDM), Verona integron-encoded metallo-β-lactamase (VIM) and Imipenmase (IMP)^14^.

C_max _and area under the curve (AUC) of ceftazidime proportionally increase with the dose; avibactam demonstrates linear pharmacokinetics across the dosage range^15^. Ceftazidime, as well as avibactam, are excreted via kidneys as unchanged drugs^15^. Less than 10% of ceftazidime and 5.7% to 8.2% of avibactam are protein bound^15^. Volumes of distribution of ceftazidime and avibactam are 17 L and 22.2 L respectively^15^.

In patients with impaired renal function, the serum half-life of ceftazidime is prolonged and a dosage adjustment is recommended^16^. Moreover, in patients with complicated intra-abdominal infections and creatinine clearance of 30-50 ml/min, ceftazidime/avibactam has been found to have lower efficacy^17^.

Adverse reactions that have been described are hypersensitivity reactions, including anaphylaxis, *Clostridium difficile-*associated diarrhea and Central Nervous System reactions, such as seizures, especially in patients with renal impairment^15^.

Ceftazidime/avibactam is indicated for the treatment of complicated intra-abdominal infections and complicated urinary tract infections caused by the following susceptible Gram-negative microorganisms: *Escherichia coli, Klebsiella pneumoniae, Proteus mirabilis, Enterobacter cloacae, Klebsiella oxytoca, Citrobacter freundii* complex and *Pseudomonas aerugin*osa, in patients 18 years or older, based on clinical trials which proved its non-inferiority when compared to carbapenems^17-19^. In complicated intra-abdominal infections, combination therapy of ceftazidime/avibactam and metronidazole is recommended^15-17^.

Recent approval for hospital-acquired bacterial pneumonia and ventilator-associated bacterial pneumonia based on the Reprove study, a phase 3, multicenter, double-blind, randomized trial which included 870 patients, make ceftazidime/avibactam a viable therapeutic option for hospital-acquired pneumonia^20^.

Ceftazidime/avibactam might be valuable for the Intensive Care Unit (ICU) patient when is also used as carbapebem-sparing antibiotic^6^. However, a retrospective case series argued the quick emergence of resistance in treated patients^21,22^.

### *2.2* *Obiltoxaximab*

**Obiltoxaximabis a monoclonal antibody directed against the protective antigen (PA) of *Bacillus anthracis*, thus preventing it from binding to cellular receptors^23^. It is a chimeric agent, consisting of enhanced 14B7V_H_ and V_L_ genes connected to human ν1 and K constants, which was derived from the murine monoclonal antibody 14B7, with mutations resulting in a 50-fold increase in affinity and corresponding neutralizing capability^24^.

Obiltoxaximab is the second monoclonal antibody approved for the treatment of inhalational anthrax, the other being raxibacumab^25^. It can be administered as pre-exposure prophylaxis, in which case it is the only therapeutic option, and in confirmed cases in combination with the appropriate antimicrobials^24^.

As clinical trials with intentional exposure of humans to anthrax are unethical, its efficacy was examined in multiple studies conducted in two animal models of inhalational anthrax, including New Zealand White rabbits (two studies) and cynomolgus macaques (4 studies) at disease onset following lethal challenge with aerosolized *Bacillus anthracis *spores^26^. In these studies, obiltoxaximab monotherapy neutralized PA and increased survival across the range of disease severity, indicating clinical benefit of toxin neutralization in both early and late stages of inhalational anthrax^26,27^.

The human dose was selected and justified by comparing observed drug exposures in animals to observed exposures in healthy and infected humans^28^. In humans at a dose of 16 mg/kg IV obiltoxaximab AUC was >2 times that in animals, while maximum serum concentrations were comparable^28^.

Obiltoxaximab has a black box warning due to severe hypersensitivity reactions that have been reported during infusion, including anaphylaxis; a premedication with diphenhydramine is recommended^23^. Although not yet approved, recent studies on humans using obiltoxaximab via intramuscular route showed good efficacy with no hypersensitivity reactions^29^.

### 2.3 *Bezlotoxumab*

**Bezlotoxumab is a fully human monoclonal IgG1 antibody directed against *Clostridium difficile *toxin B^30^. Bezlotoxumab exerts its action by impeding the binding of toxin B to colonic cells and consequently preventing development of *C. difficile* infection^31^. By neutralizing the toxin B, bezlotoxumab attenuates pro-inflammatory responses *in vitro* and reduces damage to epithelial tissue of colonic explants^32,33^. It has no direct antimicrobial activity against *C. difficile*, it has low immunogenicity and is generally well tolerated^34^. Recent data suggest that it is also cost-effective when administered together with standard-of-care antibiotics^35^.

FDA approval in October 2016 was obtained based on two double-blind, randomized, placebo-controlled, phase 3 clinical trials, Modify I and II, which involved 2655 adults treated for primary or recurrent *C. difficile *infection. In these trials, bezlotoxumab was associated with a lower rate of recurrent infection and had a safety profile similar to that of placebo^36^. Based on the population studied in the trials, it has been proposed that the risk factors justifying treatment with bezlotoxumab are: age over 65 years, history of previous *C. difficile* infection, immunosuppression and presence of virulent strain or severe *C. difficile* infection^37^.

Another monoclonal antibody, actoxumab, directed against *C. difficile *toxin A, is available^38^. Modify trials did not show any benefit in adding actoxumab to bezlotoxumab. On the contrary, the rates of sustained cure were lower compared to bezlotoxumab alone^36^.

### *2.4* *Delafloxacin***

**Delafloxacin is a new fluoroquinolone with a potential role in the treatment of acute bacterial skin and skin structure infections, in adults. It was shown to be active against Gram‐positive pathogens (*Staph. aureus, including methicillin-resistant, methicillin-susceptible isolates, Staph. haemolyticus, Staph. lugdunensis, Strep. agalactiae Strep. anginosus group and Enterococcus faecalis*) and some Gram-negative bacteria (*Escherichia coli, Enterobacter cloacae, Klebsiella pneumoniae, Pseudomonas aeruginosa*)^39^.

Its mechanism of action consists of inhibition of the activity of bacterial DNA topoisomerase IV and DNA gyrase (topoisomerase II), thus interfering with bacterial DNA replication by preventing the relaxation of positive supercoils introduced as part of the elongation process^40^. Due to its chemical structure, delafloxacin is weakly acid, unlike other fluoroquinolones, thus displaying a preserved antibacterial action through a reduced minimum inhibitory concentration in environments with low pH^40^. It is highly protein bound (84%), primarily to albumin, has a large distribution volume (48 L) and has a half-life of 3.7-8.5 hours, with a peak serum concentration of 7.45 mg/L after a 1 hour infusion^40^. Its clearance is ensured in approximately equal proportions by renal and non-renal pathways^40^.

Delafloxacin received FDA approval after demonstrating its non-inferiority to the combination of vancomycin and aztreonam in two phase 3 studies, in adult patients with acute bacterial skin and skin structure infections (PROCEED Study Group)^41-43^. Its efficacy in community-acquired bacterial pneumonia is currently under investigation in a phase III clinical trial (NCT02679573)^44^.

The drug is contraindicated in patients with known hypersensitivity to delafloxacin or any of the fluoroquinolone class of antibacterial drugs. Its common side effects include nausea, diarrhea, headache, transaminase elevations and vomiting^43^. Of note, FDA issued a black box warning related to the risk of tendinitis, tendon rupture, peripheral neuropathy, CNS effects, exacerbation of myasthenia gravis, hypersensitivity reactions and *Clostridium Difficile*-associated diarrhoea^40,43^.

Unlike other fluoroquinolones, it does not seem to elevate the risk of QTc interval prolongation on the EKG, or phototoxicity. Its use appears to be safe in patients with renal disease or hepatic impairment^40,43,45,46^.

### *2.5* *Vabomere***

**Vabomere is the first combination of a carbapenem and a β-lactamase inhibitor, consisting of meropenem, a broad spectrum carbapenem antibacterial, and vaborbactam, a β-lactamase inhibitor approved by the FDA in August 2017 for the treatment of complicated urinary infections (including pyelonephritis) in adult patients^47^.

Meropenem-susceptible microorganisms include Gram-negative bacteria like *Escherichia coli*, *Klebsiella pneumoniae*, *Enterobacter cloacae* species complex or *Pseudomonas aeruginosa*^48^. After binding to penicillin-binding proteins, meropenem inhibits the final step of peptidoglycan synthesis in bacterial cell walls and thus the biosynthesis of cell walls, leading to bacterial lysis^49^. Vaborbactam, a β-lactamase inhibitor, has no antibacterial efficacy on its own, but it blocks carbapenemases produced by *Klebsiella pneumoniae *and other β-lactamases, which could cause degradation of meropenem, thus leading to *in vitro* activity against nearly all (99%) of *Klebsiella pneumoniae* carbapenemase producing Enterobacteriaceae^49^.

Meropenem is 2% protein bound, has a distribution volume of 20.2 L and a half-life of 2.3 hours^49^. Vaborbactam is 33% protein bound, has a distribution volume of 18.6 L and a half-life of 2.2 hours^49^. While vaborbactam is not well metabolized and is excreted in the urine over a 2 day period, approximately 30% of the meropenem dose is metabolized by hydrolysis of the β-lactam ring to an inactive form, which is excreted in the urine, and between 40-60% of the meropenem dose is excreted unchanged within 2 days^49^.

Meropenem-vaborbactam received FDA approval after demonstrating its superiority over piperacilline-tazobactam for the treatment of complicated urinary tract infections, including acute pyelonephritis, in a phase 3, multicenter, randomized, double-blind, double-dummy study including 550 patients (TANGO I trial)^50^. Due to its activity against multi-drug resistant bacteria, meropenem-vaborbactam shows promising implications in the treatment of ventilator-associated pneumonia (TANGO II trial)^51,52^. A phase III, multicenter, prospective, randomized, double-blinded TANGO III trial comparing vabomere and piperacilline/tazobactam in hospital-acquired or ventilator-associated pneumonia is launced and estimated to be completed in 2010 (NCT03006679). The drug is contraindicated in patients with hypersensitivity to any of the two components or to other drugs in the same class, and the most frequently encountered adverse reactions include headache, phlebitis or infusion site reactions, and diarrhea^47^. Rare but severe side effects include hypersensitivity reactions, seizures (especially in patients treated with valproic acid), *Clostridium difficile*-associated diarrhea, thrombocytopenia, neuromotor impairment, development of drug resistant bacteria and overgrowth of nonsusceptible organisms^49^. Precautions should be taken and doses should be adapted for the patients with renal function impairment^53^.

### *2.6* *Ozenoxacin*

**Ozenoxacin, a non-fluorinated quinolone, received FDA approval in December 2017 for the topical treatment of impetigo caused by *Staphylococcus aureus* or *Streptococcus pyogenes *in adult and pediatric patients older than 2 months^54^.

The drug is bactericidal against susceptible microorganisms through inhibition of bacterial DNA replication enzymes, DNA gyrase A and topoisomerase IV^54^. After topical application, the majority of ozenoxacin plasma samples were below the limit of quantification, suggesting no systemic absorption. Thus, the distribution, metabolism and excretion of ozenoxacin have not been investigated in humans^55^.

FDA approval was granted after a phase 3 randomized, double-blind, multicenter study proved the efficacy and safety of ozenoxacin in the treatment of impetigo^56^. Adverse reactions, such as rosacea or seborrheic dermatitis, were rarely reported, but prolonged use may result in overgrowth of nonsusceptible bacteria and fungi^54^.

**Table 1:** Main characteristics of the described antibacterial drugs^15,16,23,30,31,34,43,47,53^ Food and Drug Administration (FDA), European Medicines Agency (EMA), complicated intra-abdominal infections (cIAIs), complicated urinary tract infections (cUTIs), hospital acquired bacterial pneumonia (HABP), ventilator associated bacterial pneumonia (VABP), acute bacterial skin and skin structure infections (ABSSI), intravenous (iv), hours (h), therapy (ther.).

NAME (generic/brand/ class)	Approval status	Indication	Administration	Dose and duration
Ceftazidime/avibactam / avycaz (USA), zavicefta (Europe) / combination of ceftazidime, 3rd generation cephalosporin, with avibactam, a β-lactamase inhibitor COMPANY: Allergan Inc (USA), Pfizer (Europe)	FDA: 1. since 2015 in combination with metronidazole (cIAIs cUTIs 2. since 01/02/2018 for HABP and VABP EMA: 1. since 2016 for cIAIs, cUTIs, HABP, VABP 2. infections due to aerobic Gram-negative organisms (adults - limited options)	CIAIs, cUTIs, HABP, VABP	Ceftazidime 2g and avibactam 0.5g. IV infusion over 2h	1. For cIAIs and cUTIs, ceftazidime 2g / avibactam 0.5g every 8 hours, for 5-14 days. 2. For HABP and VABP, ceftazidime 2g / avibactam 0.5g /8h for 7-14 days. 3. Adaptation of doses in case of renal function impairment.
Obiltoxaximab/ Anthim/ Monoclonal antibody COMPANY : Elusys Therapeutics	FDA: approved in March 2016 EMA: not approved	Inhalational anthrax	Diluted in 0.9% Sodium Chloride,IV infusion over 1 hour and 30 minutes	Adult patients 16 mg/kg. In paediatric patients, weight adaptation needed, greater than 40 kg - 16 mg/kg, 15 to 40 kg - 24 mg/kg, less than or equal to 15 kg - 32 mg/kg
Bezlotoxumab/ Zinplava/ Monoclonal antibody COMPANY: Merck Sharp & Dohme Limited	FDA: Approved in October 2016 EMA: Approved in January 2017	Prevention of CDI recurrence, in >18 years old patients with antibiotic ther.	Diluted solution iv infusion over 60 min using a low-protein binding 0.2-5 µm in-line or add-on filter.	Recommended dosage is 10 mg/kg. Not evaluated in patients below 18 years of age
Delafloxacin/ Baxdela/ fluoroquinolones COMPANY: Melinta Therapeutics	FDA: approved in June 2017 for ABSSI EMA: application in March 2018	ABSSI	IV infusion or oral use	300 mg/12h for 5-14 days iv infusion over 1h 450 mg/12h orally for 5 to 14 days. Renal adaptation needed.
Meropenem-Vaborbactam/ Vabomere/ combination of meropenem, and vaborbactam, a β-lactamase inhibitor COMPANY: Melinta Therapeutics	FDA: approved in August 2017 for adults with cUTI, including pyelonephritis EMA: marketing authorization application submitted in July 2017	cUTI, including pyelonephritis	Single-dose vials containing 2 g (1 g meropenem and 1 g vaborbactam) as a sterile, dry powder (iv)	4g administered over 3 hours by intravenous infusion every 8 hours for up to 14 days. Renal adaptation needed.
Ozenoxacin/ xepi/ non-fluorinated quinolone COMPANY: Medimetrics Pharm.	FDA: approved in Dec. 2017 for the treatment of impetigo Staph. Aureus, Strept. pyogenes (> 2 months old)	Impetigo	Pale-yellow 1% cream, Topical use only	Topically applications to the affected area twice a day for 5 days

## 3. Antimicrobials under investigation

Other drugs or combinations are in different stages of clinical research. World Health organization extensively reviewed, from a global health perspective, the antibacterial agents in clinical development in 2017, with focus on innovativeness and expected activity on priority pathogens^57^. Despite the total number of 33 new antibiotic entities and 9 new biological agents targeting global priority pathogens, the 7 drugs against *Mycobacterium tuberculosis* and the 9 agents against *C. difficil*e, the review noticed an insufficient “clinical pipeline” and “a lack of potential treatment options for priority resistant bacteria, especially for multidrug- and extensively drug-resistant Gram-negative pathogens”, including resistant to anti-tuberculosis treatment^57^.

A recent article by Bassetti M. et al. reviews the potential drugs in treating ventilator-associated pneumonia^51^. Beside the already FDA-approved tedizolid (2014), ceftolozane/tazobactam (2014), ceftazidime/avibactam (2015) and meropenem/vaborbactam (2017), there are studies concerning cefiderocol, imipenem/relebactam, ceftaroline/avibactam, aztreonam/avibactam, plazomicin, eravacyclin, murapavadin^51^. From this list, cefiderocol stands out, since its FDA and EMA approvals are expected in 2018^58^.

Cefiderocol, also known as S-649266, is a siderophore cephalosporine showing potency against Gram-negative bacteria^58^. A particular capacity to chelate iron makes it able to penetrate the outer bacterial membrane through bacterial iron-transporting systems^59^. A large study using clinical collections from North America and Europe evaluated the bacterial spectrum of cefiderocol and found a good activity against Gram-negative bacteria, even for resistant species, like meropenem-non susceptible *Enterobacteriaceae* (MIC <4 mcg/ml for 97% of isolates)^60^. This effect was also noted for *Pseudomonas aeruginosa* and, interestingly, for *Acinetobacter baumanni*, making this new molecule particularly valuable in the case of difficult to treat resistant infections^60^. 3 Phase III clinical trials are evaluating the potential role and safety of cefiderocol in treating carbapanem-resistant enterobacteria infections, ventilator associated pneumonia or urinary tract infections, and their results are awaited with interest (NCT02714595, NCT03032380, NCT02321800).

Recently, the future perspectives in antimicrobial research have become a little more optimistic. Description of a specific isolation chip by Nichols et al. that allows the identification of new antibiotic sources in soil microorganisms has opened the gates for new discoveries^61^. Antimicrobial discovery had been significantly slowed down by the difficulties in culturing environmental microorganisms that were concerning 99% of the species^62^. These methodological improvements lead to the discovery of new classes of antibiotics. One very promising anti-Gram-positive bacteria recently described is *teixobactin*, an 11-residue, macrocyclic depsipeptide, first identified by Ling *et al.* in 2015, which possesses a very strong inhibitory action on peptidoglycan synthesis^63^. New species of β-proteobacteria temporarily named *Eleftheria terrae* have been used during the process^63^. This molecule proved to be extremely potent in vitro against *Staphylococcus aureus, *including methicillin-resistant variants (minimal inhibitory concentration (MIC) 0.25 mcg/ml), *Mycobacterium tuberculosis *(MIC 0.125 mcg/ml), Vancomycin-resistant *Enterococcous faecium *(MIC 0.5 mcg/ml), *Clostridium Difficile* (MIC 5 ng/ml) and *Bacillus anthracis *(MIC20 ng/ml), but not on Gram-negative bacteria^63^. During this study, no resistance was observed and teixobactin shown good efficacy and tolerance in methicillin-resistant* Staphylococcus aureus* (MRSA) induced-sepsis mice models^63^.

This strong bactericidal effect can be explained by its ability of blocking the cell wall synthesis through synergistic inhibition of peptidoglycan and teichoic acid formation, by binding the precursor lipid II and lipid III, causing cell wall injury and the destruction of bacterial cell^64^. No study in humans has yet been performed and the road to clinical practice might be long, but there are hopes that this new class could be the long awaited solution for the burden of MRSA and VRE (Vancomycin-resistant* Enterococcus*) infections, as well as for the resistant strains of *Mycobacterium tuberculosis*^65^.

Another newly discovered antibiotic class using a culture-independent approach are the *malacidins*, Hover *et al*. publishing their results in 2018^66^. The malacidins have a lipopeptidic structure, with protidic core, which includes 4 non-proteinogeic aminoacids^66^. The 10 members of this class are differentiated by a methylene group at the end of the lipidic branch^66^. Malacidin A revealed a calcium-dependent bactericidal *in vitro* and *in vivo* effect on Gram-positive bacteria like *Staphylococcus aureus,* including vancomycin-resistant variants. No resistance was detected^66^. As in the case of teixobactin, the potential clinical benefit is significant and the evolution of the studies on malacidins is followed with a great interest.

## 4. Conclusion

The last 3 years brought new antimicrobial drugs available for clinical use. Some agents like obiltoxaximab, bezlotoxumab antibodies and ozenoxacin target narrow areas of interest. Future studies and clinical practice will define the place of delafloxacin among the other acute skin infection treatments, and likewise concerning the role of the new combinations - ceftazidime/avibactam and meropenem/vaborbactam - in the practical management resistant bacterial infections in the Intensive Care Unit. The recent discovery of new antibiotic classes and the augmentation of the source pool for further research have brought a glimmer of optimism. But the road to actual clinical benefit might be long and the past experience has taught us that resistance can develop even for very promising molecules. A shared and vigorous research effort is continuously needed in order to improve the therapeutic options for the increasingly resistant and highly adaptable germs.
